# Myxœdema

**Published:** 1890-05-24

**Authors:** 


### The Practitioner's Mirror and Retrospect.

*[The Editor will be glad to receive offers of co-operation and contributions from members of the profession. There is no doubt that much valuable material full of interest and instruction is at present lost to science. This is largely so in the case of (1) the younger and less-known members of the hospital staff (2) those connected with cottage hospitals, and (3) the great body of medical practitioners not attached to any institution. The co-operation of all is cordially invited to enable the Editor to make* The Hospital *of the utmost possible use and value to the profession. Specialists willing to review books on their special subjects are invited to communicate this fact at once. All letters should be addressed to* The Editor, The Lodge, Porchester Square, London, W.]

### MEDICINE.

### MYXŒDEMA.

This name we believe was invented by Dr. William Ord from the Greek terms signifying mucus and swelling, and Dr. Ord defined the malady as a progressive disease in which the tissues of the body are invaded by jelly-like mucus-yielding dropsy, unaccompanied by albuminuria, or other signs of primary affection of the kidneys. But we imagine that it was a paper by Sir W. Gull, read at a meeting of the Clinical Society in 1873, which first attracted notice to the malady, when Sir W. Gull described a cretinoid state supervening in adult life in women. Charcot, however, proposed calling the disease *cachexiæ pachydermique*, which has not found favour in this country, at least. Although a good deal has been already written on the subject, there is still considerable obscurity attaching to it. The disease in its various forms into which some have divided it, viz., primary myxœdema, cachexia strumepriva, and sporadic cretinism, has been held to depend upon, or to be associated with, loss of the thyroid gland. In 1883 Senior brought to notice the similarity of the symptoms of the maladies mentioned above. And Reverdin, of Geneva, followed with cases in which the thyroid gland having been removed for goitre, there ensued symptoms resembling myxœdema. In 1888 a report of a committee of the London Clinical Society appeared, in which it is stated that the influence of locality and of social position is apparent; that tubercle is frequently associated, but that neither alcohol nor syphilis seem to have a part in the production of myxœdema. The typical signs of myxœdema are increase of general bulk and swelling of the skin, which does not pit, and which is not affected by gravitation. There is also dryness and roughness. It occurs, perhaps, most frequently in the supraclavicular regions. There is also turgescence of the mucus membrane of the mouth, remarkable physiognomy, slow, painful, monotonous utterance, slowness of thought and movement, and impaired memory. There is also tendency to fall, owing to disorder of co-ordination, and often absence or shrinking of the thyroid gland, which becomes of a yellow colour. Microscopically, changes in the thyroid are described as cell infiltration of the walls of the vesicles, followed by epithelial proliferation in the vessels. At a later date the gland becomes converted into a delicate fibrous tissue, in which remains of vesicles are scattered. The function of the thyroid gland is supposed by Horsley to be concerned with hæmatopoiesis, not only of the corpuscles, but of the other elements of the blood. But, as a matter of fact, we do not know what are the functions of either the thymus or the thyroid body. At least half-a-dozen theories have been formed regarding the latter. Some have endeavoured to surmount the difficulty by supposing that the thyroid, together with some other so-called ductless glands, are the remains of organs which once were of importance in the economy, but which in the process of evolution have come to play only a very subsidiary part. At present we are scarcely in a position to define the relation between the dwindling of the thyroid gland and the condition we have, in this country at least, agreed to term myxœdema, for cases have occurred in which the thyroid, although small, had not disappeared. Dr. Mitchell Clarke, physician to the Bristol General Hospital, has recently described a typical case of the disease. A woman, aged 51, who had borne nine children, became the subject of myxœdema. It appears there was hæmorrhage after the birth of the last child, and the patient had not been strong since, suffering considerably from megrims. The climateric period occurred at 37. She commenced to suffer from weakness, drowsiness, nervousness, giddiness, insomnia at night, and the appearance changed. The features became dull and heavy, the face pallid, the eyelids œdematous, but with a waxy rather than soft feel. The voice became hoarse, the hair brittle, the nose broad and clumsy. There was also puffiness about the ankles and desquamation of the cuticle of the hands and feet, but the fingers were not "spade-shaped," a term first applied by Sir W. Gull. The tongue was large, and there were patches of swelling in the mouth. No thyroid could be felt, and as the patient was not fat the region could be well explored. The thoracic organs were healthy. There was no albumen in the urine. Sensation was perfect. The mental state was usually placid, but the woman was irritable and suspicious, and the speech slow and drawling. She also had a delusion that there was constantly someone under her bed. Myxœdema generally presents in women, but not invariably, for some time back Dr. Saville exhibited at the Medical Society a male patient afflicted with the disease. It usually occurs at or after middle age, but not always, for Dr. Abercrombie, in November last, brought a patient before the Clinical Society, in whom the disease commenced at the age of eight or nine. When myxœdema was first recognised, Dr. Mahomed argued in favour of its being identical with Bright's disease, but at the present time no one holds this view. Sir Andrew Clark regards the malady as curable by careful diet, arsenic, iron, friction, and baths. It has also been reported that under the use of jaborandi considerable improvement has been noticed. Sweating and salivation may be induced by a full dose of jaborandi, otherwise the physiological action of the drug does not appear to promise much benefit.

There is another malady which somewhat resembles myxœdema, which has been termed *Acromegaly.* It was first described by Marie in 1866, and has been investigated by Virchow and others. Acromegaly affects particularly the hands, feet, and neighbouring parts. The hands become thick, fat, and paw-like. The face is involved to a less degree. The thickening is due to an increase of the *panniculus adiposus*, and in this respect it appears to resemble myxœdema. But in the latter the bones are not involved. The recognition of this is, however, often difficult from the great thickening of the soft parts. And the majority of cases hitherto described have shown marked decrease of muscular power which gradually leads to spinal curvature. No connection has been traced to the presence or absence of the thyroid. Virchow suggested that heredity is an element in the production of acromegaly.

